# Genetic Diversity of the Cestode *Echinococcus multilocularis* in Red Foxes at a Continental Scale in Europe

**DOI:** 10.1371/journal.pntd.0000452

**Published:** 2009-06-09

**Authors:** Jenny Knapp, Jean-Mathieu Bart, Patrick Giraudoux, Marie-Louise Glowatzki, Isabelle Breyer, Francis Raoul, Peter Deplazes, Georg Duscher, Karel Martinek, Pavol Dubinsky, Marie-Hélène Guislain, Florence Cliquet, Thomas Romig, Andrzej Malczewski, Bruno Gottstein, Renaud Piarroux

**Affiliations:** 1 Institute of Parasitology, Vetsuisse Faculty, University of Bern, Bern, Switzerland; 2 Department of Chrono-Environment, CNRS 6249, usc INRA, University of Franche-Comté, Besançon, France; 3 Department of Clinical Research, Vetsuisse Faculty, University of Bern, Bern, Switzerland; 4 Institute of Parasitology, University of Zurich, Zurich, Switzerland; 5 Institute for Parasitology and Zoology, Department for Pathobiology, University of Veterinary Medicine, Vienna, Austria; 6 Department of Biology, University of West Bohemia, Pilsen, Czech Republic; 7 Parasitological Institute, Slovak Academy of Sciences, Kosice, Slovak Republic; 8 2C2A-CERFE, Boult-aux-Bois, France; 9 AFSSA, Malzéville, France; 10 Institute of Zoology, University of Hohenheim, Hohenheim, Germany; 11 Witold Stefanski Institute of Parasitology, Polish Academy of Sciences, Warszawa, Poland; 12 Department of Parasitology and Mycology, Hôpital la Timone, Marseille, France; Queensland Institute of Medical Research, Australia

## Abstract

**Background:**

Alveolar echinococcosis (AE) is a severe helminth disease affecting humans, which is caused by the fox tapeworm *Echinococcus multilocularis*. AE represents a serious public health issue in larger regions of China, Siberia, and other regions in Asia. In Europe, a significant increase in prevalence since the 1990s is not only affecting the historically documented endemic area north of the Alps but more recently also neighbouring regions previously not known to be endemic. The genetic diversity of the parasite population and respective distribution in Europe have now been investigated in view of generating a fine-tuned map of parasite variants occurring in Europe. This approach may serve as a model to study the parasite at a worldwide level.

**Methodology/Principal Findings:**

The genetic diversity of *E. multilocularis* was assessed based upon the tandemly repeated microsatellite marker EmsB in association with matching fox host geographical positions. Our study demonstrated a higher genetic diversity in the endemic areas north of the Alps when compared to other areas.

**Conclusions/Significance:**

The study of the spatial distribution of *E. multilocularis* in Europe, based on 32 genetic clusters, suggests that Europe can be considered as a unique global focus of *E. multilocularis*, which can be schematically drawn as a central core located in Switzerland and Jura Swabe flanked by neighbouring regions where the parasite exhibits a lower genetic diversity. The transmission of the parasite into peripheral regions is governed by a “mainland–island” system. Moreover, the presence of similar genetic profiles in both zones indicated a founder event.

## Author Summary

*Echinococcus multilocularis* is a tapeworm of the red fox, which represents a considerable health threat to respectively infected humans. Main endemic areas are located in China, Siberia, and central Europe. Alarmed by an emerging or reemerging situation in Europe, the question of how the parasite gets spatially and temporally spread and transmitted becomes essential to prepare appropriate control programs. The question was tackled by using genetic data on a large sample size of *E. multilocularis* adult stage tapeworms, combined with geographical site location data input. The historically documented endemic area, represented by the northern Alpine arch, was shown to harbour the highest genetic richness and diversity, as compared to surrounding areas in northern and eastern Europe. The spatial and temporal spread of different *E. multilocularis* genotypes in Europe seems to be ruled by a founder event, linked to exportation of parasites from the central core to newly identified (western and eastern) areas or subregions, where these parasites could subsequently disseminate under geographical separation from the original foci.

## Introduction

An increasing number of studies use genetic markers to reconstitute the epidemiological history of human and veterinary diseases and identify underlying environmental factors of the spread of the pathogens involved [1],[2]. Most of these studies focused on vector-borne diseases while the remaining addressed zoonotic bacterial or viral diseases. Parasitic diseases caused by helminths are common worldwide; conversely to microorganisms, their life cycle and transmission patterns are very complex, because they frequently include several developmental stages and also different hosts and are thus largely dependent on multiple environmental factors. For tapeworms, the involvement of definitive and intermediate hosts, which both have their own environmental requirements regarding their habitat, results in a complex set of interactions between host and parasite populations that finally drives the spatial distribution of the parasite.

The present study investigated the spatial distribution and respective spread of the helminth *Echinococcus multilocularis* by studying its genetic diversity at a continental scale. This parasite has a wide geographic distribution in the northern hemisphere, the endemic area stretching from North America through Europe to central and east Asia and includes northern parts of Japan. Humans and intermediate host animals (mostly small rodents) acquire the infection by ingesting *E. multilocularis* eggs via contaminated food or water or through close physical contact with infected foxes, dogs, or feces of these hosts. In humans, the resulting alveolar echinococcosis (AE) is a highly lethal disease caused by the larval stage of the parasite. AE primarily affects the liver by inducing a proliferative hepatic disorder mimicking that of liver cancer. The prevalence of AE did not regress with the improvement of health in developed/industrialized countries. Furthermore, ecological changes during the past two decades have resulted in a significant increase of the European fox population, together with a marked urbanization phenomenon of this wild carnivore [3]. Thus, wild foxes especially in urban and periurban areas are in close contact with a high-density human population and represent an emerging health risk to residents of European countries if a high prevalence of *E. multilocularis* is maintained. Recent database analyses spanning the past 50 years suggested that the annual incidence of human AE in Switzerland increased significantly [4]. Similar developments have been reported in many other European countries. Furthermore, since the beginning of the 1990s human AE cases have now also been registered in eastern European countries, including Poland [5], Slovakia [6], and Lithuania [7], with sporadic incidences in some other countries in eastern and southern Europe.

Three hypotheses can be proposed to explain the basic mechanisms responsible for the spread and expansion of *E. multilocularis* in Europe and for the recent detection of the disease (AE) outside the previously recognised endemic areas. In the first hypothesis, European areas endemic for echinococcosis constitute a single focus where the dispersal movement of foxes has led to the establishment of a single and homogeneous population of parasites covering a large part of the continent. In such a case, the recently identified eastern and western zones have long existed but were detected only recently due to active searching as a consequence of increased disease awareness. The second hypothesis proposes that the endemic areas in Europe were composed of a set of foci located in western, central, and eastern Europe, in which the parasite populations remained more or less isolated. In this case, similarity or discontinuity within the parasite populations can be observed, depending respectively on the presence or absence of genetic exchanges between the different foci that had existed for a long time. Similarly to the first two hypotheses, the seeming emergence of AE in eastern Europe can be interpreted as a result of a better awareness and thus identification of cases in humans (and animals). The third hypothesis states that the suspected spread of *E. multilocularis* in Europe has been governed by a “mainland–island” system of parasite transmission. In such a system, the ancestral focus in central Europe acted as mainland and supplied the peripheral European areas, thanks to the dispersal movement of foxes [8]. From a genetic point of view, this mainland–island hypothesis should be supported by the presence of similar genotypes in western, central, and eastern Europe associated with an imbalance of genetic diversity between parasites in the ancestral versus recent endemic foci as a result of one parasite population invading the new territory and therefore exporting its genetic profile. This phenomenon could be attributed to a founder event, where rare species or genotypes in the primary focus could become dominant in a newly colonized area [9].

Investigations on the transmission pattern and dynamics of *E. multilocularis* in Europe are considered as a public health challenge for a rare but lethal infection presently threatening European countries. Thus, a genetic investigation carried out on adult stage hermaphroditic *E. multilocularis* parasitizing the red fox in Europe, combined with respective information on geographical localisation, should contribute to better understanding of the epidemiology of this parasite. Genetic studies based on the analysis of classical coding and noncoding DNA targets had demonstrated earlier a striking homogeneity among *E. multilocularis* specimens isolated from different geographical regions [10],[11]. As a consequence, a more sensitive genetic tool with a high power of discrimination was generated, which is based on microsatellite targets represented by tandemly repeated noncoding nuclear sequences. Whereas single locus microsatellite *E. multilocularis* sequences allowed only the description of genetic polymorphism at large-scale spatial levels [12],[13], the tandemly repeated multilocus microsatellite EmsB [14] yields a sensitivity that enables the detection of genetic diversity at regional and local geographical scales [13],[15]. On the basis of the experience accumulated with EmsB so far, we undertook an international study, involving several European countries, in order to provide a better understanding of the epidemiology of *E. multilocularis* in Europe, with special emphasis on wildlife transmission of the parasite within fox populations. The major aims of the study were (i) to address the genetic profiles of *E. multilocularis* on a continental scale investigating parasite samples from seven European countries and (ii) to compare the genetic diversity between parasite populations in the historically recognised endemic area versus newly identified eastern and western zones.

## Materials and Methods

### Collection of *E. multilocularis* samples

**Figure 1:**
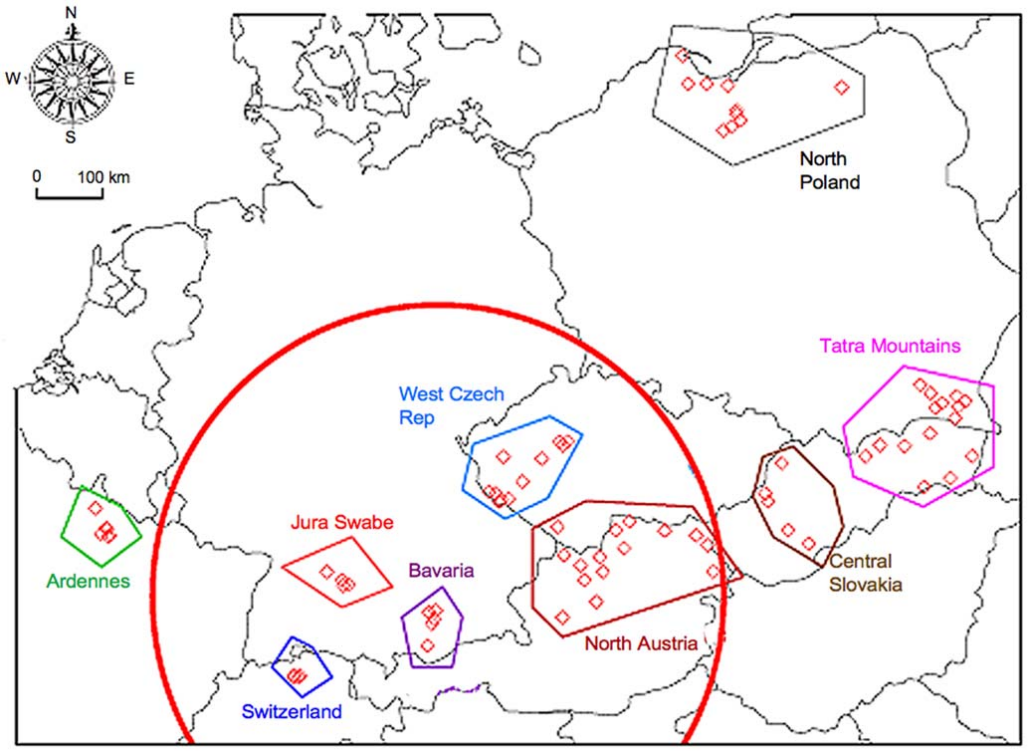
Spatial distribution of *E. multilocularis* sample collection. Red lozenges represent the position of fox; polygons define a geographically restricted subregion. The red circle area indicates the historically documented *E. multilocularis* central endemic focus in Europe.

The study was designed to collect five individual worms per fox intestine and 20 foxes per geographical area investigated (for geographical areas, refer to Table 1). The final panel obtained was composed of 571 worms originating from 123 autopsied red foxes, allocated into nine subregions based on topographical and ecological criteria (Figure 1). Collections were performed by nine research units dealing with diagnostic and epidemiological aspects of *E. multilocularis* in Europe (see author list and respective information). Collection and worm isolation procedures were carried out identically in all laboratories according to a standard protocol [16] using the intestinal scraping technique (IST) as described by Deplazes and Eckert [17]. Worm specimens were individually preserved in 70% (v/v) ethanol until use. Thus, the subregion of Ardennes demarcated with polygons clustered 79 individual adult stage *E. multilocularis* samples (derived from 16 foxes), Switzerland 84 (19 foxes), Jura Swabe 39 (8 foxes), Bavaria 48 (10 foxes), west Czech Republic 61 (13 foxes), north Austria 103 (23 foxes), central Slovakia 30 (7 foxes), Tatra Mountains (east Slovakia and south Poland) 81 (17 foxes), and north Poland 46 (10 foxes). Among these subregions, Switzerland, Jura Swabe, Bavaria, west Czech Republic, and north Austria were gathered into a set of the historical endemic area, whereas the others were considered as western (Ardennes) and eastern (central Slovakia, Tatra Mountains, and north Poland) European subregions. Parasites were collected between 2001 and 2005.

**Table 1:** Sample size of each EmsB profile per subregion.

	Subregions										
	Historical Endemic Area					Western Area	Eastern Areas				
EmsB Profiles	Swiss	NorthAu	Swab	Bav	WestCz	Arde	NorthPol	Tatras	Cent Slov	No. of Worms	No. of Subregions with the Profile
G01	0	0	0	0	0	0	6	0	0	6	1
G02	0	0	0	0	0	3	0	1	0	4	2
G03	0	0	0	0	0	2	1	0	0	3	2
G04	2	0	2	0	3	0	0	0	0	7	3
G05	0	29	**10**	0	**26**	12	0	9	4	90	6
G06	0	0	0	0	5	0	0	0	0	5	1
G07	3	10	8	5	0	0	**38**	5	0	69	6
G08	0	0	0	0	0	0	1	0	0	1	1
G09	0	0	0	0	1	0	0	0	0	1	1
G10	5	0	0	0	1	1	0	0	0	7	3
G11	3	0	0	0	0	0	0	0	0	3	1
G12	1	0	0	0	0	0	0	0	0	1	1
G13	1	0	0	0	0	0	0	0	0	1	1
G14	1	0	0	0	0	0	0	0	0	1	1
G15	**16**	0	0	0	0	0	0	0	0	16	1
G16	1	0	0	0	0	0	0	0	0	1	1
G17	0	0	1	6	0	0	0	0	0	7	2
G18	0	5	2	5	0	0	0	0	0	12	3
G19	0	0	5	0	12	0	0	0	0	17	2
G20	6	4	0	0	5	0	0	0	0	15	3
G21	4	14	5	0	5	0	0	9	0	37	5
G22	1	2	0	0	0	18	0	0	0	21	3
G23	11	**31**	2	0	7	0	0	10	**25**	86	6
G24	5	0	0	0	0	0	0	0	0	5	1
G25	0	0	4	0	0	0	0	4	0	8	2
G26	0	0	0	0	0	**43**	0	0	0	43	1
G27	6	0	0	0	0	0	0	2	0	8	2
G28	1	4	0	**25**	0	0	0	**41**	0	71	4
G29	8	0	0	0	0	0	0	0	0	8	1
G30	5	0	0	0	0	0	0	0	0	5	1
G31	4	0	0	7	0	0	0	0	0	11	2
G32	0	0	0	0	0	0	0	0	1	1	1
No. worms (No. foxes)	84 (19)	103 (23)	39 (8)	48 (10)	61 (13)	79 (16)	46 (10)	81 (17)	30 (7)	571 (123)	
Total of EmsB profiles	*19*	*8*	*9*	*5*	*9*	*6*	*4*	*8*	*3*		

Swiss, Switzerland, Canton of Zurich; NorthAu, north Austria; Swab, Jura Swab; Bav, Bavaria; Arde, Ardennes; WestCz, west Czech Republic; NorthPol, north Poland; EastSlovPol, east Slovakia and south Poland; CentSlov, central Slovakia. Bold numbers indicate the genotype with the highest number of worms per subregion. No of worms: total number of worms collected from all foxes obtained from a respective region (maximum five worms per fox).

### Geographical localisation and ecological gathering of samples

For each area, red foxes were obtained from licensed hunters or governmentally employed rangers as hunting or road casualties. The animals were individually labeled with an identification number and information on the exact locality, date, and cause of death. For German, French, Polish, Slovak, and Austrian foxes, the name of the next approximate locality was taken as geographical reference. For Swiss and Czech foxes, the geographical position was recorded by local teams with the help of a Global Positioning System apparatus. The Swiss sample positions were recorded in the Swiss Coordinate System CH1903. Geographical coordinates of Czech foxes were transformed to the World Geodesic system (WGS84) with the help of MapInfo software version 6.5. Coordinates of the centers of the locality districts were used as the geographical coordinates. The Swiss coordinates were transformed to WGS84 format with the help of the Federal Office of Topography website (http://www.swisstopo.ch). All coordinates finally were transformed into UTM projected system tile 31 on which Euclidian distances were computed (see below). Because samples were collected in different parts of countries, isolates were geographically separated into different subregions of origin based on topographical and ecological criteria, with the help of R software 2.6.1 and packages sp, rgdal, foreign, maptools, and splancs (http://cran.r-project.org/).

### DNA extraction from tapeworms

Total genomic DNA was isolated and purified from single adult-stage worm specimens using a DNA Easy Tissue Kit (QIAGEN, Switzerland). After a washing step with bidistilled water, the worms were subsequently stored in 70% (v/v) ethanol. DNA extraction was carried out according to the manufacturer's protocol. Purified DNA was eluted in 200 µl of elution buffer (provided by the manufacturer) and stored at −20°C until use.

### PCR and fragment size analysis

The genetic diversity of *E. multilocularis* was assessed by fluorescent PCR, followed by fragment size analysis with the tandem repeated multilocus microsatellite target EmsB (GenBank accession number AY680860). This target is composed of two repeated and juxtaposed motifs: (CA)*_i_* and (GA)*_j_*, where *i* and *j* are the number of repetitions. This microsatellite pattern is repeatedly integrated into the parasite DNA, exhibiting polymorphism between the different loci [14]. The variations in motif composition and the number of fragment copies allowed the identification of different profiles [13],[14]. PCR was carried out in a 30-µl reaction mixture containing 20 to 50 ng of purified DNA of a whole single worm, 200 µM of each dNTP (GeneAmp dNTP, Applied Biosystems, Foster City, CA), 0.4 µM of fluorescent forward primer, 5′-labeled with specific fluorescence dye (EmsB A*: 5′Fam-GTGTGGATGAGTGTGCCATC-3′), 0.7 µM of classical reverse primer (EmsB C: 5′-CCACCTTCCCTACTGCAATC-3′), and 0.5 U of AmpliTaq DNA polymerase enzyme, associated with GeneAmp 1× PCR Buffer (10 mM Tris-HCl, pH 8.3, 50 mM KCl, 1.5 mM MgCl_2_, and 0.001% gelatin) (Applied Biosystems, Foster City, CA, USA). The amplification reaction was performed in a Biometra thermocycler (Whatman Biometra, Goettingen, Germany), under the following conditions: a pre-amplification step at 94°C for 13 min, followed by 45 cycles with denaturing at 94°C for 30 s, annealing at 60°C for 30 s, and extension at 72°C for 1 min, and for the final elongation of DNA strands an ending extension at 72°C for 45 min. Reproducibility of the EmsB amplification was ensured by performing each PCR reaction two times under identical conditions. The size polymorphism of PCR products was determined by using an automatic sequencer (ABI Prism 3100; Applied Biosystems, Foster City, CA). Fluorescence signals generated by incorporated dye primers into PCR products were detected by colorimetric analysis. Correspondences were automatically established to assess the sizes of the amplified fragments by the help of the Genotyper 3.7 software.

### Genotyping of *E. multilocularis*

The electrophoregrams of the EmsB target are presented as a series of peaks that correspond to alleles. The presence of peaks and their associated heights were recorded as previously described [14],[15]. These data were used to assess genetic diversity and to establish clusters or assemblage profiles by hierarchical clustering analysis, using the Euclidean distance and the unweighted pair group method with arithmetic mean [18]. Stability of clusters was tested by a multiscale bootstrap resampling (*B* = 1,000), resulting in approximately unbiased *p*-values [19],[20]. Clustering analyses were performed by using the R software 2.6.1 [21] and package pvclust [22]. A genetic distance threshold, previously reported by Knapp et al. [13], was applied to the dendrogram to describe clusters among the sample collection. This threshold was calculated by means of three samples of the unique strain F AUB-2, maintained in vivo in *Meriones unguiculatus* between March 1991 and February 1992 [14]. As previously described, the genetic distance threshold at 0.08 enabled us to define assemblage profiles for samples clustered under this limit [13],[15]. The stability of this approach had already been documented previously by using also a 7-year term of serial transplantation of a metacestode isolate kept within *M. unguiculatus* [14].

### Richness and diversity analyses of EmsB profiles

Rarefaction analyses were undertaken in each subregion to assess whether sampling allowed accurate estimations of genetic richness (number of EmsB profiles) and diversity (inverse Simpson index reflecting the number and relative abundance of EmsB profiles). Rarefaction curves represent the number of profiles (or the diversity index) as a function of sampling effort (i.e., number of foxes). When the curves reach an asymptote, one can therefore assume that sampling pressure was enough to obtain a reasonable estimation of the parameters [23]. The analyses were performed using EstimateS software version 7.5 [24].

### Investigations on the geographical structure of genetic diversity

The geographical distribution of each EmsB profile is illustrated on a European map (see below). The question of genetic isolation by geographical distance was investigated; a hypothesis was tested on the comparison of the Euclidean distance between EmsB profiles and the geographical distances between samples. This hypothesis was tested with the help of the Mantel test, based on a comparison between two distance matrices [25], using R software 2.6.1 and the package pgirmess [26].

## Results

### *E. multilocularis* genetic diversity settlement in the European area of investigation

**Figure 2:**
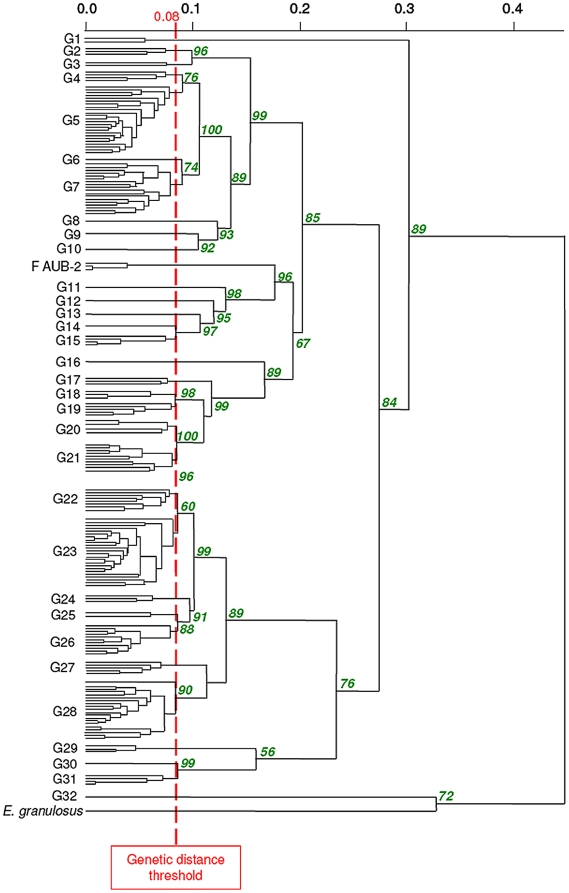
Dendrogram constructed from EmsB amplification data, achieved by hierarchical clustering analysis (Euclidean distance and unweighted pair group method). Details of sample composition of the EmsB profiles G01 to G32 are given in Table 1. Approximately unbiased *p*-values are indicated in italics and in percent at each node of the tree, calculated with multiscale bootstrap (*B* = 1000). Worms from the same fox showing a genetic distance of less than 0.025 were pooled to simplify the dendrogram. F AUB-2: referring to three independent samples obtained from an *E. multilocularis* reference strains, maintained *in vivo* in *Meriones unguiculatus* during 7 months; these samples were taken to establish the genetic distance threshold of 0.08. An outgroup control is represented by *E. granulosus* sensu stricto (G1 strain, originating from an Algerian sheep [35]).

EmsB profiles were represented by series of peaks as previously described [13],[14]. Reproducibility of amplification of the EmsB microsatellite target (Pearson correlation coefficient, *r* = 0.88 to 0.99, *p*<0.01) was in agreement with previous studies [13]. EmsB profiles were clustered in a dendrogram using a method and criteria that were previously described [13]. Briefly, two EmsB electropherogram profiles were included in the same cluster when the differences between their electrophoretic profiles were strictly below the 0.08 threshold that was defined by stability experiments (as detailed in [13]). Finally, 32 clusters of EmsB profiles were defined at the European level, named G1 to G32 (Figure 2); each cluster included 1 to 90 worm samples.

On the basis of a selection of Swiss, French, south Polish, and east Slovakian (Tatra Mountains) foxes (*n* = 86) from which five individual worms had been isolated, 56 (65%) animals harboured worms belonging to a single cluster, whereas 30 foxes (35%) harboured mixed infections (worms belonging to two or three clusters).

**Figure 3:**
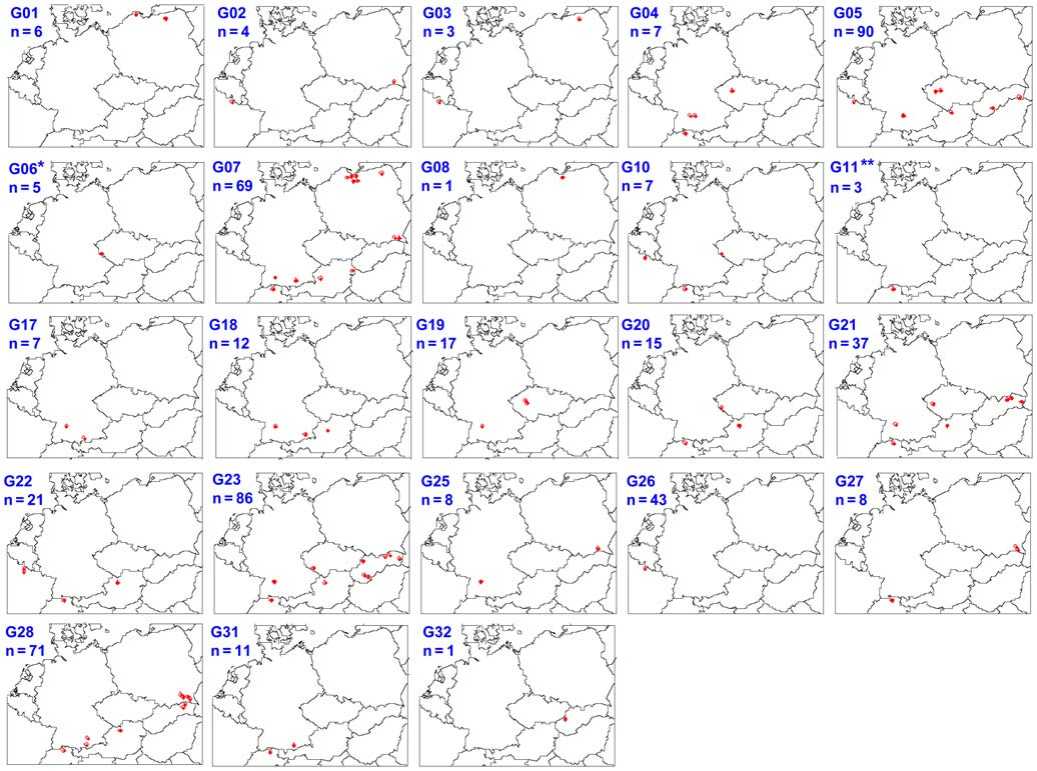
Distribution of EmsB profiles in the nine subregions in Europe. For each profile, the red dots represent the position of one or more red foxes; *n* corresponds to the total number of worms found with the respective profile. Single localisations of different profiles were represented in a single map for **Switzerland: G12 (1), G13 (1), G14 (1), G15 (16), G16 (1), G24 (5), G29 (6), G30 (5) and *Czech Republic: G9 (1); these data are not shown in this figure.

The distribution of clusters (also named EmsB profiles) in subregions is shown in Table 1 and graphically presented on maps in Figure 3. Six clusters were found to be numerically dominant among all samples (in order of relative abundance G05, G23, G28, G07, G26, and G21 in Table 1). Each of the 6 clusters included between 37 and 90 worms. Altogether, these six clusters represented 69% of the whole collection, and except for G26, they were widely distributed over the whole area of investigation. For example, the G05 cluster was found in north Austria, Jura Swabe, Ardennes, west Czech Republic, Tatra Mountains, and central Slovakia (*n* = 90 worms). Despite its wide area of distribution, the electrophoregrams representing this cluster were remarkably similar: respective genetic distance values between the isolates yielded an average of 0.039 and a standard deviation of 0.0123. The distribution of the frequencies of each cluster by subregion showed differences between central and peripheral subregions of our panel. In the four western and eastern subregions, a single cluster of profiles (G23 in central Slovakia, G28 in Tatra Mountains, G07 in north Poland, and G26 in Ardennes, respectively) accounted for more than half of the samples (83%, 51%, 83%, and 54%, respectively), whereas only one central subregion, Bavaria, exhibited a dominant cluster (G28, 52%) (Table 1). Cluster G26 was only found in the Ardennes subregion. Finally, seven clusters were represented by only a single worm (G08, G09, G12, G13, G14, G16, and G32 in Table 1), among the total of 571 worms collected. These clusters were predominantly localised in the centre of the whole study area.

### Geographical structure of genetic diversity

**Figure 4:**
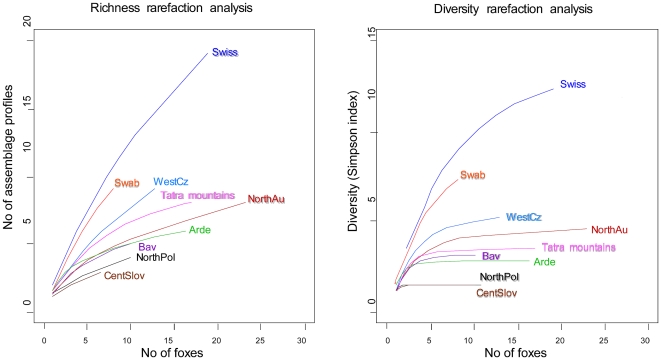
Genetic richness and diversity (inverse Simpson index) rarefaction analysis curves for each subregion (fox as a sampling unit). Swiss, Switzerland, canton of Zurich; NorthAu, north Austria; Swab, Jura Swab; Bav, Bavaria; Arde, Ardennes; WestCz, west Czech Republic; NorthPol, north Poland; EastSlovPol, east Slovakia and south Poland; CentSlov, central Slovakia.

Richness and diversity rarefaction analyses were carried out for each geographical group of foxes (Figure 4A and 4B). Globally, the number of different clusters was underestimated in every subregion (richness rarefaction curve did not reach a plateau). Moreover, because sampling pressure differed among subregions, richness was compared among subregions with a number of foxes equivalent to that of the least sampled subregion (i.e., seven foxes in central Slovakia). Two groups might be distinguished: Swiss and Jura Swabe with higher richness (9.62 and 8.25) and the other subregions with lower richness (from 3 to 5.99). In contrast, genotype diversity seemed to be assessed with a reasonable accuracy (except maybe for Jura Swabe). This means that the structure of the genotype population is correctly estimated even with low sample size and underestimation of the genotype richness. Diversity was higher in Switzerland and in Jura Swabe, lower in north Poland and central Slovakia, and intermediate in the other subregions.

**Figure 5:**
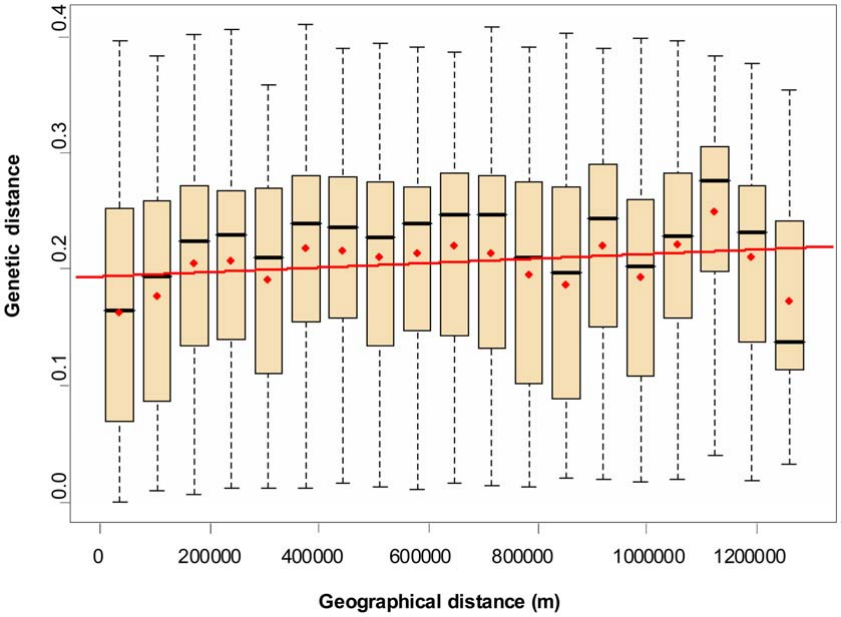
Comparison of genetic and geographical distances as assessed by the Mantel test. In each box plot, the red dot represents the genetic distance average, and the black bar the median value. The upper extremity of the box represents the third quartile, and the lower the first quartile. The dotted lines show ranges between upper and lower extreme genetic values. Upper bars represent the ninth decile, and the lower the first decile. The red line represents the regression line. Number of pairwise comparisons per class box plot from left to right: 1, 14,333; 2, 5,519; 3, 13,120; 4, 13,986; 5, 8,497; 6, 17,878; 7, 10,419; 8, 12,273; 9, 12,231; 10, 11,149; 11, 11,562; 12, 6,036; 13, 4,954; 14, 5,359; 15, 6,165; 16, 7,976; 17, 4,296; 18, 2,163; 19, 5,250.

A comparative analysis between geographical position and genetic data of each worm was carried out with 162,735 pairs of worms; findings were graphically organized into class box plots (Figure 5). The hypothesis of isolation by distance was tested by the Mantel test [25]. On the basis of 1,000 replicates, the correlation coefficient value was *r* = 0.077 (*p*<0.001). The genetic distance between samples was almost not affected by an increasing geographical distance. Indeed analysis of variance (ANOVA) statistical investigation with the linear regression model of genetic against geographical distance explained only 5‰ of variability.

## Discussion

Increased knowledge of the genetic diversity of *E. multilocularis* is a prerequisite to elucidate basic biological and population characteristics of the parasite, and a better understanding of *E. multilocularis* population dynamics is fundamental to design new strategies for control and surveillance. Because *E. multilocularis* appears to be genetically very conservative [10],[11],[27], we decided to use a more sensitive genetic marker, the microsatellite EmsB, which allows genotypic fine-tuning within this parasite species [13]–[15]. The analysis of the genetic relationship between *E. multilocularis* samples, carried out with the help of the EmsB multilocus microsatellite, yielded a total of 32 clusters of profiles within the European *E. multilocularis* collection (571 worms from 123 red foxes isolated in nine ecological European subregions). The spatial distribution of these 32 clusters (some of them being found concomitantly in the same foxes) suggests that Europe is a unique global focus of *E. multilocularis*. This focus can be schematized as a central core located in Switzerland and Jura Swabe flanked by bordering areas where the spread of the parasite is governed by a mainland–island system.

The comparison of profiles' diversity and richness between the nine studied areas showed that the populations of parasites were not homogeneously distributed in Europe. On the one hand, Switzerland and Jura Swabe, both known as historically documented endemic regions, exhibited the highest genetic diversity of the parasite population compared with those of the other areas. On the other hand, in Slovakia and Poland, where human AE cases have only recently been registered, the genetic diversity of the parasite was the lowest. A very similar unbalanced distribution of genetic diversity has also been shown in studies dealing with other parasites, such as *Leishmania tropica*, where the historically recognised endemic area also colocalised conclusively with the region exhibiting the highest diversity [28]. The imbalance of genetic diversity between the central (ancestral) and peripheral areas and the presence of distinct clusters of profiles in peripheral areas similar to those in the central European area support the hypothesis that the whole *E. multilocularis* focus in Europe is governed by a mainland–island system of parasite transmission. Thus, ancestral foci supplied hitherto nonendemic areas by dispersal based on fox mobility and migration [8]. This imbalance of genetic diversity between ancestral and recently identified endemic areas could be attributed to the founder effect, establishment with a small number of individuals, which have brought a part of the genetic diversity from the historically documented endemic area to the newly identified (western and eastern) areas or subregions [9]. The founder event is also supported by the findings that in the peripheral but not in the central region a single cluster of profiles was prevalent and that the seven genotypes represented by only one worm were predominantly localised in the central area. Results also showed that Switzerland and Jura Swabe exhibited a higher genetic diversity than the other foci of the historically documented endemic central Europe, suggesting that the recent expansion to the four peripheral subregions could have been preceded by a first expansion from Switzerland and Jura Swabe to Bavaria, west Czech Republic, and north Austria. Another driving factor, such as the parasite biomass (or intensity of transmission), also could be partially responsible for the differences in genetic diversity observed in this study. For example, in the Zurich urban area, a high-density fox population of 10 foxes per km^2^ has been registered, with a parasite prevalence of approximately 40–60% and with high numbers of infected rodents found within this area [3]. Therefore, the total biomass of such a sampled area is very high, increasing the probability of a high diversity at variable genetic loci.

Among the 32 EmsB clusters of profiles, five represented 62% of the whole European collection and were widely distributed (Figure 3). These clusters were found throughout Europe, indicating that the whole zone should be considered as a single focus, where the dispersal movement of foxes allowed for the spreading of the parasites from one country to another within a time period short enough to avoid a substantial genetic drift. The weak effect of the geographical distance on the distribution of profiles is reinforced by the comparison of genetic and geographical distance matrices, assessed by the Mantel test and ANOVA, which indicated that the geographical distance is only a minor factor among those involved in the genetic distribution of *E. multilocularis* in Europe. Thus, to obtain an overview of the genetic epidemiology of *E. multilocularis*, it is mandatory to operate at the continental level.

Although the timescale associated with the dissemination of *E. multilocularis* in Europe remains unclear, the rather widespread distribution of clusters indicates an almost well established dynamic circulation of the parasite in Europe. Recently, a mathematical model was used to assess the migration of *E. multilocularis* from an endemic to a noncontaminated area in the Netherlands [29]. As an example, in this region, the parasite was predicted to progress at the speed of 2.7 km per year from the northern part to the southern part of the country. Consequently, in the case of a founder event, an EmsB profile that is simultaneously found in countries separated at broad distances (e.g., G07 found in Switzerland and Poland) must have been dispersed by foxes at least many decades ago. This is in contradiction with the hypothesis that the recent description of human cases in eastern Europe over the last few decades was due to the concomitant spread of the parasite in the Czech Republic and in Poland. Even if eastern European areas can be considered as foci that have been relatively recently colonized by *E. multilocularis*, the parasite needed more than a few decades to migrate distances of more than 1,000 km. Thus, in those countries the apparent emergence of human AE is more likely due to an active search as a consequence of disease awareness and only secondarily due to an increase of parasite prevalence.

In western Europe, the presence of area-specific clusters in the French Ardennes indicated that a particular genotype has been isolated for many generations and progressively differed from the other genotypes circulating in the neighbouring areas by, for example, a genetic drift. A recent study conducted in the Alpine watershed in the north of Italy (Val Pusteria area) indicated that the Alpine natural barrier could isolate parasite populations, which progressively became genetically different [30]. In the case of the French Ardennes, this differentiation could not be explained by any geographical obstacle, because other clusters, such as the common G05 cluster, are also present in the Ardennes. One could speculate that differences in the life cycle (especially concerning intermediate host species) of the parasite in the Ardennes as compared to those in the neighbouring areas could have led to this genetic drift [31]. So far, we know that in the Ardennes the main rodent involved as an intermediate host is *Microtus arvalis* [32], whereas in neighbouring areas such as the Jura massif of France, Switzerland, and southern Germany include both *Arvicola terrestris* and *M. arvalis* [33],[34]. However, this question will be the subject of more detailed analyses in future investigations. Without the evidence presented above, the findings on the French Ardennes also support our second hypothesis, stating that the endemic areas in Europe are composed of a set of foci in which the parasite populations remained more or less isolated.

Apart from the main data discussed above, our study confirms the previous works of Nakao and co-workers [12] and Knapp and co-workers [15], showing the simultaneous presence of *E. multilocularis* adult stage worms of different genotypes in the same intestine of a single fox. Here, we show that this phenomenon occurs frequently, with 35% of foxes harbouring worms belonging to more than one cluster of profiles in all areas that we investigated. The origin of this phenomenon may be intense predation activity of definitive hosts [12],[13] or the presence of mixed infection already in an intermediate host, thus resulting in ingestion of protoscoleces belonging to several clusters However, mating events are rare within *E. multilocularis* individuals, as indicated by previous studies carried out with other molecular tools [15],[27].

In conclusion, this is the first study systematically addressing the genetic diversity of a helminth population on a continental scale. This large-scale focus, however, is close to the adjacent Russian–Siberian endemic zone, which may also contribute to the genetic diversity in Europe, or vice versa. This point has not yet been addressed in the present study but will deserve attention in future investigations addressing the global genetic diversity of *E. multilocularis*. Nevertheless, by application of EmsB profiles as tools for the genetic characterisation of *E. multilocularis* in Europe, hypotheses could be formulated on the pattern of migration and dissemination of the parasite covering a whole continent. These hypotheses should be confirmed in the future, with similar studies including parasite samples from intermediate hosts (rodents and humans) to complete the data presented here. In addition, the genotyping of the parasite involved in human AE could allow us to trace back the history of the infection, which is one of the main challenges in epidemiological studies of AE.
